# Complete Resection of a Giant Middle Mediastinal Castleman Disease Using Robot-Assisted Thoracic Surgery: A Case Report

**DOI:** 10.70352/scrj.cr.26-0040

**Published:** 2026-09-10

**Authors:** Hiroki Imabayashi, Yuki Sata, Takahide Toyoda, Terunaga Inage, Kazuhisa Tanaka, Junichi Morimoto, Masako Chiyo, Yukiko Matsui, Masayuki Ota, Jun-ichiro Ikeda, Hidemi Suzuki

**Affiliations:** 1 Department of General Thoracic Surgery, Chiba University Graduate School of Medicine, Chiba, Chiba, Japan; 2 Department of Diagnostic Pathology, Chiba University Graduate School of Medicine, Chiba, Chiba, Japan

**Keywords:** Castleman disease, middle mediastinal tumor, robot-assisted thoracic surgery, case report

## Abstract

**INTRODUCTION:**

Castleman disease is a rare benign lymphoproliferative disorder characterized by lymph node hyperplasia. Unicentric Castleman disease (UCD) involves a single anatomical region. Complete surgical resection remains the standard treatment. Lesions in the middle mediastinum can become large and technically demanding because of their proximity to the trachea, esophagus, and major vessels. Although video-assisted thoracoscopic surgery has been used in selected cases, reports of robot-assisted thoracic surgery (RATS) for giant UCD arising in the subcarinal middle mediastinum remain extremely limited. Herein, we report a case illustrating the feasibility and potential advantages of RATS in the treatment of giant middle mediastinal UCD.

**CASE PRESENTATION:**

A man in his 40s was incidentally found to have a 9.2 × 6.4 cm middle mediastinal mass beneath the tracheal bifurcation on chest imaging. The patient was asymptomatic, and laboratory tests, including interleukin-6 and tumor markers, yielded normal results. Fluorodeoxyglucose PET/CT demonstrated a homogeneous lesion with mild uptake. Endobronchial US-guided transbronchial needle aspiration was nondiagnostic, and upper gastrointestinal endoscopy showed no direct invasion of the esophagus. Although UCD was suspected, the differential diagnoses included malignant lymphoma and a solitary fibrous tumor. Complete surgical resection was planned for both diagnosis and treatment because of the uncertain diagnosis, the tumor size, and its location. The tumor was mobilized and removed en bloc using right-sided RATS with 4 robotic ports and 1 assistant port. The robotic platform provided 3D visualization, articulated instrumentation, and precise dissection, particularly around the right main bronchus, esophagus, azygos vein, and posterior aspect of the tumor. The operative time was 311 min, and the estimated blood loss was 200 mL, with no major intraoperative bleeding or postoperative complications. Histopathology confirmed hyaline-vascular-type UCD, and margin-negative resection was achieved. The postoperative recovery was uneventful, and the patient was discharged on POD 5.

**CONCLUSIONS:**

When combined with meticulous preoperative planning and strategic port placement, RATS may offer a feasible minimally invasive approach for managing large mediastinal tumors in challenging locations.

## Abbreviations

CD
: Castleman disease
RATS
: robot-assisted thoracic surgery
UCD
: unicentric Castleman disease
VATS
: video-assisted thoracoscopic surgery

## INTRODUCTION

CD is a rare lymphoproliferative disorder first described by Castleman as a thymoma-like mediastinal tumor.^1)^ It is classified into UCD, which is confined to a single anatomical region, and multicentric CD, which involves multiple lymph node stations.^2,3)^ UCD often presents as a solitary mass in the abdomen, neck, or mediastinum. Complete surgical resection serves both diagnostic and curative purposes in the management of UCD.^4)^ We report a rare case of giant middle mediastinal UCD located beneath the tracheal bifurcation that was completely resected using RATS. Although VATS has been used in selected cases,^5–7)^ reports of RATS for giant UCD arising in the subcarinal middle mediastinum remain extremely limited. This case highlights the feasibility and technical utility of RATS for the complete resection of a giant UCD in this anatomically challenging location. This case report was prepared in accordance with the CARE reporting checklist.^8)^

## CASE PRESENTATION

**Fig. 1:**
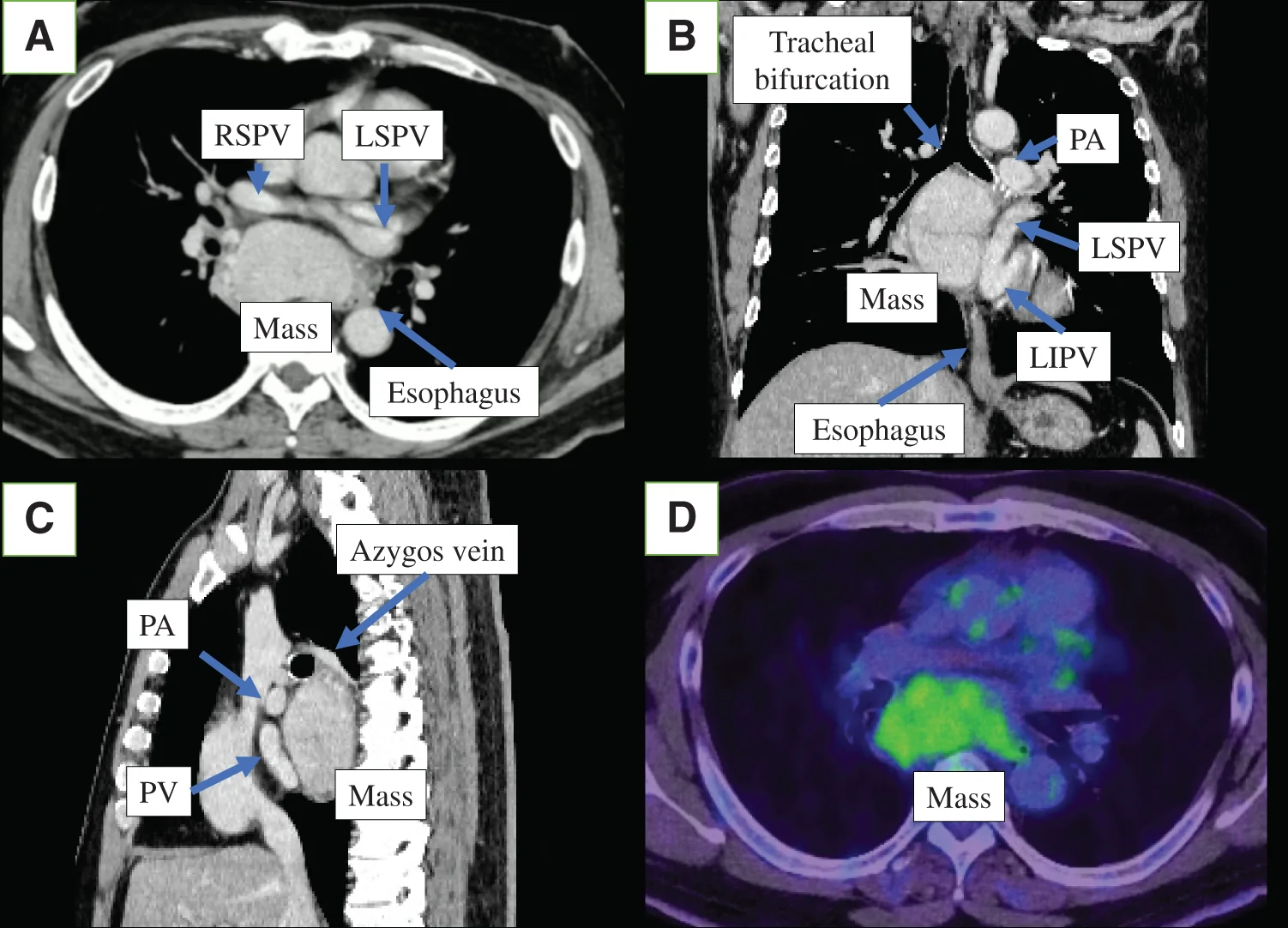
Preoperative imaging findings. (**A**) Axial contrast-enhanced CT showing a 9.2 × 6.4 cm middle mediastinal mass beneath the tracheal bifurcation. (**B**) Coronal contrast-enhanced CT showing the relationship between the tumor and adjacent structures, including the tracheal bifurcation and azygos vein. (**C**) Sagittal contrast-enhanced CT showing the subcarinal location of the tumor and its proximity to the esophagus and major mediastinal structures. (**D**) PET/CT showing mild uptake, with a maximum standardized uptake value of 3.9. LIPV, left inferior pulmonary vein; LSPV, left superior pulmonary vein; PA, pulmonary artery; PV, pulmonary vein; RSPV, right superior pulmonary vein; SPV, superior pulmonary vein

**Fig. 2:**
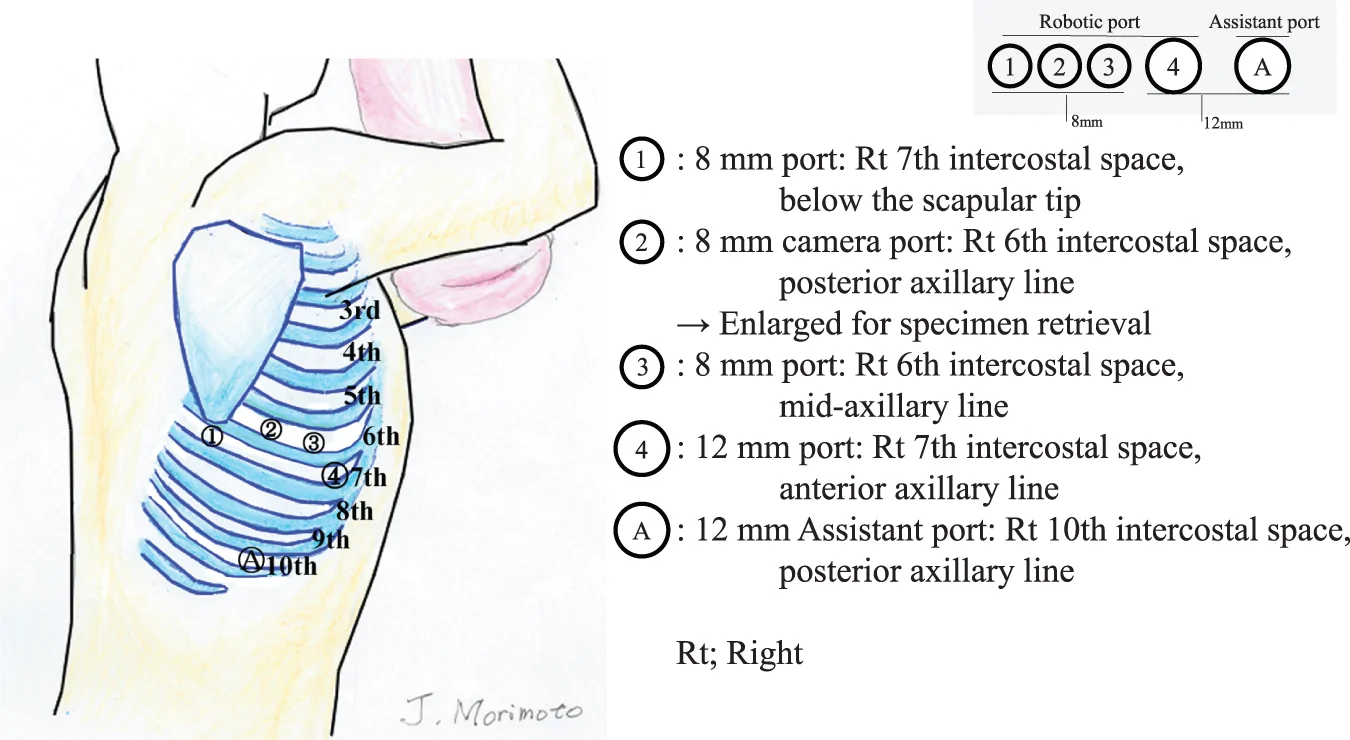
Port configuration. 1: 8-mm port: right 7th intercostal space, below the scapular tip. 2: 8-mm camera port: right 6th intercostal space, posterior axillary line → Enlarged for specimen retrieval. 3: 8-mm port: right 6th intercostal space, mid-axillary line. 4: 12-mm port: right 7th intercostal space, anterior axillary line. A: 12-mm Assistant port: right 10th intercostal space, posterior axillary line.

**Fig. 3:**
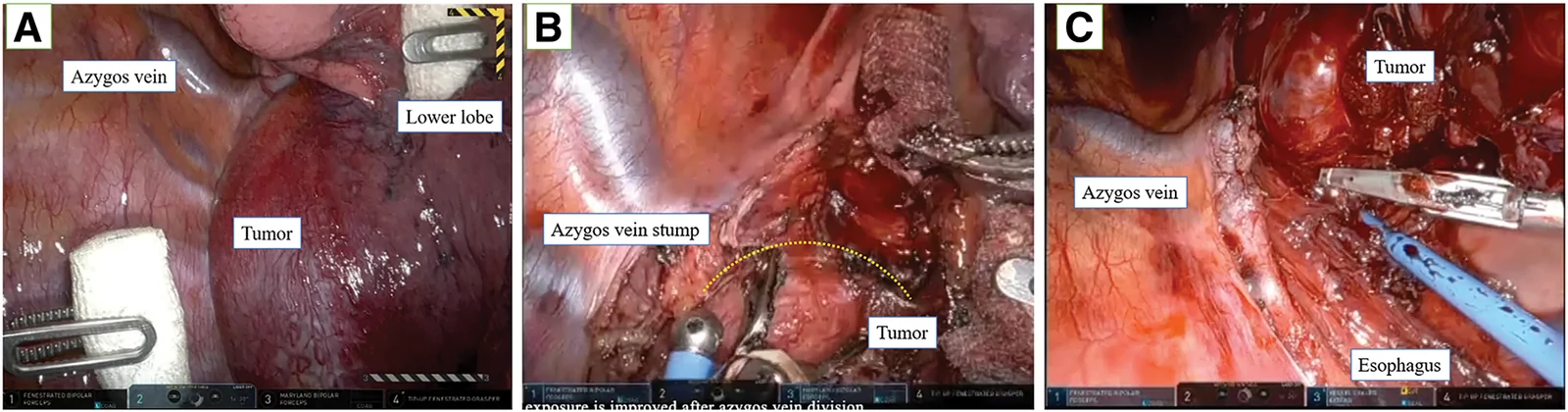
Intraoperative findings. (**A**) The tumor in the middle mediastinum was surrounded by prominent neovascularization. (**B**) After division of the azygos vein, the cranial aspect of the tumor extended to the dorsal side of the azygos vein. (**C**) After division of the azygos vein, the posterior aspect of the tumor was clearly visualized during dissection along the esophageal side.

**Fig. 4:**
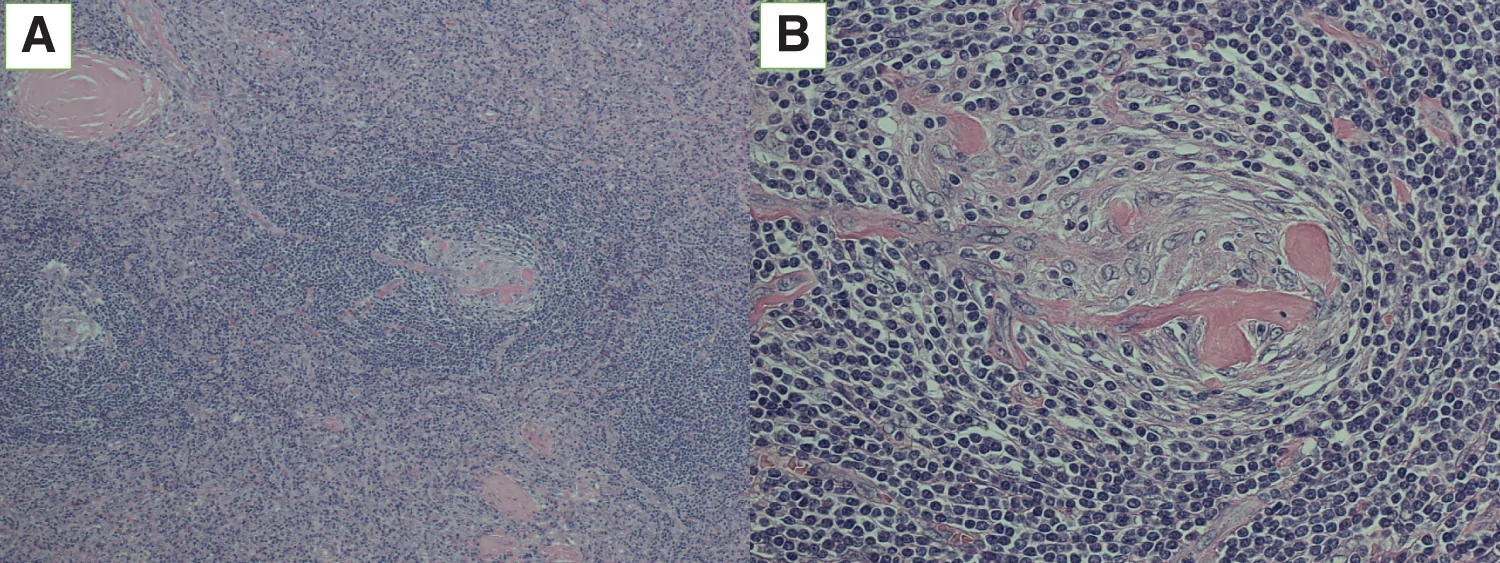
Histopathological findings. (**A**) Low-power view of a hematoxylin and eosin-stained section shows lymphoid follicles of various sizes. Original magnification, ×10. (**B**) High-power view shows an atrophic germinal center penetrated by a hyalinized vessel, creating a lollipop-like appearance. Original magnification, ×40.

A man in his 40s with a medical history of dyslipidemia and ulcerative colitis was referred to our hospital after an abnormal shadow was incidentally detected on a chest radiograph. The patient was asymptomatic at presentation. His regular medications included fenofibrate, mesalazine, and butyrate-producing bacterial preparations. There was no relevant family or psychosocial history related to the present condition. The physical examination findings were unremarkable. Contrast-enhanced CT revealed a 9.2 × 6.4 cm mass in the middle mediastinum beneath the tracheal bifurcation, adjacent to the esophagus, right main bronchus, azygos vein, pulmonary artery, and pulmonary vein (**Fig. 1A**–**1C**). Laboratory investigations showed that tumor markers and interleukin-6 levels were within normal limits. MRI demonstrated a homogeneous solid mass, whereas PET/CT showed mild uptake, with a maximum standardized uptake value of 3.9 (**Fig. 1D**). Endobronchial US-guided transbronchial needle aspiration yielded a limited specimen containing blood components, cartilage tissue, and a very small number of atypical epithelioid cells with enlarged nuclei and eosinophilic cytoplasm. Because of the limited amount of diagnostic tissue, the findings were insufficient to determine whether the lesion was benign or malignant. Upper gastrointestinal endoscopy showed no intraluminal exposure of the tumor or direct invasion of the esophagus, although mild extrinsic compression was suspected. Accordingly, complete surgical resection was planned as both a diagnostic and therapeutic intervention. Because preoperative imaging showed no evident direct invasion but demonstrated close proximity to vital mediastinal structures, we planned complete resection using a right-sided robotic approach to obtain adequate exposure of the subcarinal space. The port placement was designed to provide access from the inferior pulmonary ligament to the cranial aspect of the subcarinal space. Division of the azygos vein and limited lung resection were considered preoperatively as possible maneuvers to secure exposure and avoid bronchial injury, but the final decisions were made intraoperatively. A single 12-mm robotic port was placed as the fourth robotic port to allow the use of a robotic stapler when necessary, and a 12-mm assistant port was also placed at the posterior axillary line in the tenth intercostal space (**Fig. 2**). The camera port was an 8 mm port throughout the robotic procedure and was enlarged only at the end of the operation for specimen retrieval. A brief edited intraoperative video is available in **Supplementary Video 1**. The surgery was performed using the da Vinci Xi Surgical System (Intuitive Surgical, Sunnyvale, CA, USA) under general anesthesia with 1-lung ventilation in the left lateral decubitus position. Four robotic ports and 1 assistant port were placed on the right side, as illustrated in **Fig. 2**. A 30° robotic endoscope was inserted through an 8-mm camera port placed at the posterior axillary line in the sixth intercostal space, and carbon dioxide insufflation was maintained at 10 mmHg. The tumor was located in the middle mediastinum, surrounded by dense neovascularization, and firmly adherent to the right lower lobe (**Fig. 3A**). Dissection was initiated by dividing the inferior pulmonary ligament and proceeded dorsally under stable robotic visualization, with careful preservation of the esophagus. The adhesion between the tumor and right lower lobe parenchyma was dissected using a Vessel Sealer Extend (Intuitive Surgical), with careful attention to avoid lung and bronchial injury. The cranial aspect of the tumor extended posterior to the azygos vein. Because the azygos vein limited exposure of the cranial aspect of the tumor, it was divided using an EndoWrist Stapler 30 with a white reload (Intuitive Surgical) through the fourth robotic port, allowing further dissection while maintaining awareness of the esophageal course and right main bronchus (**Fig. 3B**, **3C**). Although preoperative CT showed the posterior aspect of the tumor in contact with the descending aorta, robotic retraction provided excellent exposure of the posterior surface of the tumor, allowing safe dissection. As a result, the descending aorta was never directly visualized. The tumor was removed en bloc. A small pericardial defect accidentally occurred during dissection, although no firm adhesion between the tumor and the pericardium was observed. An underwater air leak test demonstrated no air leakage, and no major intraoperative bleeding occurred. The operation lasted 311 min, and the estimated blood loss was 200 mL. Histopathological examination with hematoxylin and eosin staining revealed lymphocytic proliferation with minimal atypia and diffusely scattered lymphoid follicles of various sizes. The germinal centers were atrophic, with penetrating hyalinized vessels, capillary proliferation, fibroblastic proliferation, and collagen deposition. Based on these findings, the lesion was diagnosed as hyaline-vascular-type UCD (**Fig. 4A**, **4B**). Complete resection was histologically confirmed. At the site where the tumor–lung adhesion was divided using a vessel sealer, no lung tissue was found attached to the tumor, indicating that the adhesion was dissected without injury to the lung parenchyma. The postoperative course was uneventful, and the patient was discharged on POD 5. At 9 months after surgery, the patient remained asymptomatic without evidence of recurrence. The clinical course is summarized in **Supplementary Table 1**.

## DISCUSSION

UCD is a rare lymphoproliferative disorder that can occur in the mediastinum. Although intrathoracic lymph nodes are a recognized site of involvement, UCD arising in the subcarinal middle mediastinum is uncommon and presents significant operative challenges because of the narrow anatomical space and proximity to vital structures.^9)^ In the review by Keller et al., only 3 of 81 cases involved the middle mediastinum, underscoring the rarity of this location.^10)^

Complete surgical resection remains the standard treatment for UCD and also provides a definitive diagnosis.^4,11)^ However, the optimal surgical approach should be selected according to tumor size, location, vascularity, and the relationship with adjacent organs. For tumors located near the main bronchi, esophagus, and major vessels, conventional VATS may be limited by restricted instrument angulation and visualization in the deep mediastinum. In such cases, thoracotomy may be selected to obtain adequate exposure, although it is more invasive than minimally invasive approaches.^7)^ As summarized in **Table 1**, the present tumor was among the largest reported middle mediastinal UCDs treated surgically, yet complete resection was achieved without preoperative arterial embolization or conversion to thoracotomy. Previous reports suggest that paratracheal UCD can often be managed with VATS,^12–16)^ whereas subcarinal lesions frequently require a right-sided approach to obtain adequate exposure. In larger tumors, limited visualization and difficult access may necessitate thoracotomy.^17)^

**Table 1:** Summary of reported cases of middle mediastinal UCD

Reference number	Year	Age/Sex	Tumor location	Tumor size	Approach	Number of ports	Pathology	Preoperative arterial embolization
This case	2025	40s/M	Subcarinal lymph node (#7)	9.2 × 6.4 cm	Right RATS	5	HV	No
^9)^	2015	23/M	Subcarinal lymph node (#7)	8 cm	Left posterolateral thoracotomy	NA	HV	Yes
^16)^	2015	25/F	Right lower paratracheal lymph node (#4R)	6 × 4 × 4 cm	Right VATS	3	PC	No
^14)^	2014	55/F	Right lower paratracheal lymph node (#4R)	5 × 3 cm	Right VATS with a 3.5-cm utility incision	1	HV	No
^6)^	2013	30/F	Subcarinal lymph node (#7)	NA	VATS	NA	HV	Yes
^15)^	2007	42/F	Subcarinal lymph node (#7)	7.0 × 5.5 × 4.4 cm	Right hybrid VATS with a 10-cm thoracotomy	3	HV	No

F, female; HV, hyaline-vascular-type Castleman disease; M, male; NA, not available; PC, plasma-cell-type Castleman disease; UCD, unicentric Castleman disease; RATS, robot-assisted thoracic surgery; VATS, video-assisted thoracic surgery

In the present case, the tumor measured 9.2 cm and occupied the subcarinal middle mediastinum, adjacent to the right main bronchus, esophagus, azygos vein, pulmonary vessels, and descending aorta. Therefore, the main technical requirements were adequate exposure of the subcarinal space, stable visualization in a deep operative field, and precise dissection around vital structures. RATS was selected to meet these requirements while maintaining a minimally invasive approach. In addition, modification of the standard right-sided robotic lower lobectomy port configuration^18)^ enabled caudal-to-cranial dissection from the inferior pulmonary ligament toward the subcarinal space. This port strategy was useful for approaching both the posterior and cranial aspects of the tumor while maintaining an appropriate operative view.

The intraoperative findings further illustrate the case-specific advantages of RATS. As shown in **Fig. 3A**, the tumor was surrounded by dense neovascularization and was firmly adherent to the right lower lobe. In this setting, the magnified 3D view and articulated instruments were useful for stepwise vascular control and precise dissection in the narrow subcarinal space. The cranial aspect of the tumor extended posterior to the azygos vein, and division of the azygos vein improved exposure of this portion of the tumor. The robotic view shown in **Fig. 3B** and **3C** allowed dissection to proceed while maintaining awareness of the course of the esophagus and right main bronchus. These features may be particularly advantageous in the middle mediastinum, where rigid instruments and limited viewing angles in conventional VATS can make dissection around the bronchus, esophagus, and vascular structures technically demanding.

Preoperative embolization is another important consideration for large hypervascular mediastinal tumors. Previous reports suggest that embolization can reduce intraoperative bleeding in selected cases of CD.^6,9,19)^ In the present case, contrast-enhanced CT showed multiple small feeding vessels but did not identify a dominant feeding artery suitable for selective embolization. After discussion at our departmental surgical conference, we concluded that preoperative embolization was unlikely to provide sufficient benefit. Therefore, we planned stepwise intraoperative vascular control under robotic visualization. Dense neovascularization was encountered intraoperatively, but small vessels were carefully sealed or divided under a magnified 3D view, and major bleeding did not occur. Although preoperative embolization was not performed, the possibility of conversion to thoracotomy in the event of uncontrolled bleeding had been anticipated. Nevertheless, preoperative embolization should be considered in selected cases with marked hypervascularity, a dominant feeding artery, or a high risk of uncontrolled bleeding.

Intraoperative decision-making is also important when dense adhesion to vital structures or unexpected tumor invasion is encountered. In the present case, preoperative endobronchial US and upper gastrointestinal endoscopy showed no evidence of obvious tumor invasion into the esophageal or bronchial wall. However, if safe dissection had not been feasible, conversion to an open approach, combined resection with reconstruction, or subtotal resection would have been considered according to the intraoperative findings. These considerations are important because complete resection is desirable for UCD, but surgical safety should be prioritized when firm adhesion or invasion of vital mediastinal structures is encountered.

Reports of RATS for giant UCD arising in the subcarinal middle mediastinum remain extremely limited. The present case suggests that, in selected patients, RATS with careful preoperative planning and optimized port placement may provide a feasible minimally invasive option for large UCDs in the subcarinal middle mediastinum.

## CONCLUSIONS

Herein, we report a case of hyaline-vascular-type UCD that was successfully resected using RATS. Even for a large tumor in the middle mediastinum, careful planning and optimized port placement may facilitate safe and complete resection using a minimally invasive robotic approach.

## SUPPLEMENTARY MATERIALS

Video 1 Intraoperative summary of robot-assisted tumor resection
The video shows port placement and robotic arm positioning, identification of the tumor, dissection from the pulmonary ligament side, preservation of the esophagus, dissection along the pericardial side, the operative field before division of the azygos vein, division of the azygos vein using a robotic stapler, improved exposure after azygos vein division, further cranial dissection in a deep plane, visualization of the relationship between the tumor and adjacent right lung, en bloc tumor removal, and the final operative view after resection.

Table 1 Clinical timeline

## DECLARATIONS

### Availability of data and materials

Data sharing is not applicable to this article because the datasets were neither generated nor analyzed for the case report.
