# Supplementary material for: Complete Resection of a Giant Middle Mediastinal Castleman Disease Using Robot-Assisted Thoracic Surgery: A Case Report
Source: Surg Case Rep. 2026 Sep 10;12(1):26-0040. doi: 10.70352/scrj.cr.26-0040 (PMC13585486; doi:10.70352/scrj.cr.26-0040)
Supplement: Supplementary file 1 [file scr-12-01-26-0040-s002.docx]

**Supplementary Table 1.** Clinical timeline

| Time point | Clinical event |
| --- | --- |
| Initial presentation | An abnormal shadow was incidentally detected on chest radiography. The patient was asymptomatic. |
| Preoperative evaluation | Contrast-enhanced computed tomography revealed a 9.2 × 6.4 cm middle mediastinal mass beneath the tracheal bifurcation. |
| Further imaging | Magnetic resonance imaging showed a homogeneous solid mass, and fluorodeoxyglucose positron emission tomography/computed tomography showed mild uptake with a maximum standardized uptake value of 3.9. |
| Tissue sampling | Endobronchial ultrasound-guided transbronchial needle aspiration yielded a limited specimen and was nondiagnostic. |
| Esophageal assessment | Upper gastrointestinal endoscopy showed no intraluminal exposure of the tumor or direct invasion of the esophagus. |
| Surgical treatment | Complete surgical resection was performed using right-sided robot-assisted thoracic surgery with four robotic ports and one assistant port. |
| Postoperative day 5 | The postoperative course was uneventful, and the patient was discharged. |
| Pathological diagnosis | Histopathological examination confirmed hyaline-vascular-type unicentric Castleman disease with complete resection. |
| 9 months after surgery | The patient remained asymptomatic without evidence of recurrence. |
